# Management of jugular bulb injury during retrosigmoid transmeatal resection of vestibular schwannoma

**DOI:** 10.3171/2021.7.FOCVID2155

**Published:** 2021-10-01

**Authors:** Felipe Constanzo, Bernardo Correa de Almeida Teixeira, Mauricio Coelho Neto, Ricardo Ramina

**Affiliations:** 1 Department of Skull Base Surgery, Clinica Bio Bio, Concepcion;; 2 Department of Neurological Surgery, Hospital Clinico Regional de Concepcion, Chile; and; Departments of 3 Neuroradiology and; 4 Neurological Surgery, Neurological Institute of Curitiba, Brazil

**Keywords:** jugular bulb, vestibular schwannoma, skull base surgery, retrosigmoid approach

## Abstract

Inadvertent laceration of the jugular bulb is a potentially serious complication of the retrosigmoid transmeatal approach to vestibular schwannomas. Here, the authors present the case of a 51-year-old woman with a right Hannover T4a vestibular schwannoma and bilateral high-riding jugular bulb, which was opened during drilling of the internal auditory canal (IAC). They highlight the immediate management of this complication, technical nuances for closing the defect without occluding the jugular bulb, and modifications of the standard technique needed to continue surgical resection.

The video can be found here: https://stream.cadmore.media/r10.3171/2021.7.FOCVID2155

## Transcript

### 0:27 Case Presentation

The patient was a 51-year-old woman with history of right-sided deafness first noticed 3 years prior to consult. Audiogram confirmed nonserviceable hearing on the right side, and MRI showed a cerebellopontine angle mass compatible with a Hannover T4a vestibular schwannoma extending into the most lateral part of the internal auditory canal. Bilateral high-riding jugular bulbs were also noted, reaching up to the level of the inferior limit of the IAC, with the right side being dominant. However, it did not appear as it would be exposed during drilling of the IAC.

### 1:05 Positioning and Retrosigmoid Approach

We performed a right retrosigmoid craniotomy in dorsal decubitus in standard fashion. After opening the lateral cerebellomedullary cistern and exposing the tumor, we raised a dural flap to expose the posterior wall of the IAC before beginning tumor resection.

### 1:22 Drilling of the IAC and Jugular Bulb Laceration

Drilling began using a large cutting drill bit to outline the IAC, then using a smaller bit in the lateral portion. At approximately 8 mm from the internal auditory meatus, brisk bleeding was encountered from the jugular bulb while using a large diamond bit. The head was immediately lowered at the level of the heart, and we let blood fill the surgical site, not suctioning directly over the jugular bulb, to prevent air embolism. A small cotton pledget was placed over the defect to aspirate the pooled blood safely; then a small crushed muscle patch was used to seal the defect, carefully positioning it below the cotton pledget. This graft is always obtained during the approach to reconstruct the IAC after resection of the tumor, which is why we prefer it over other synthetic materials such as bone wax or gelatin sponge. When positioning the graft, extreme care must be taken to avoid occluding the jugular bulb, so the patch must be gently placed to cover the laceration of the bulb without tamponading the lumen, nor entering the bulb and potentially become embolic material. It may take a while to obtain adequate closure and still allow proper visualization to continue the surgery. After bleeding stopped, fibrin glue was applied to seal the defect.

### 2:41 Expanded Opening of the IAC

Using cutting and coarse diamond drill bits of decreasing size, drilling was continued with care to avoid catching the muscle with the drill. Since visualization to the most lateral and anterior part of the IAC was still partially limited by the graft, we needed to expand the opening of the superior wall of the canal to improve the angle of attack. Here, we also needed to trim the muscle graft to improve visualization. Then, we continued gradually drilling around the muscle patch from medial to lateral, and from superior to inferior, palpating with a blunt dissector the limits of the canal, until we achieved sufficient exposition, which meant opening around 100° of it, far more than it is usually needed.

### 3:38 Intracanalicular Tumor Resection

The dura of the IAC was incised parallel to the path of the nerves, and then internal debulking began at the level of the meatus. Using blunt and sharp dissection, the tumor was gradually reduced and separated from the brainstem, following the arachnoid plane. Inside the IAC, the tumor was gently dissected from the nerves. In this case, the superior vestibular nerve was first identified and cut. At this point, it was noted that residual tumor remained in the most lateral part of the inferior vestibular nerve, which was the nerve of origin, so additional drilling was needed to expose the remaining lesion. By carefully drilling below the patch, the tumor was removed completely from the IAC.

### 4:19 Cisternal Tumor Resection

Lastly, the cisternal portion of the tumor was resected, first identifying the cochlear nerve in the anteroinferior quadrant of the tumor, and the facial nerve in the medial part of it. By gently dissecting the tumor from the facial nerve, gross-total resection was achieved. The facial nerve could be preserved both anatomically and functionally, whereas the cochlear nerve had to be sacrificed. Finally, the IAC was reconstructed with a large muscle patch and fibrin glue.

### 4:54 Postoperative Course

Postoperatively, the patient developed House-Brackmann grade III facial palsy and moderate headache, that spontaneously resolved on postoperative day 5. MRI confirmed gross-total resection of the lesion, as well as a small thrombus on the dome of the jugular bulb, without occlusion of venous blood flow. We do not routinely anticoagulate such cases.

### 5:16 Conclusions

In conclusion, a high-riding jugular bulb must be actively looked on preoperative images, as it may be present in around 9% of patients undergoing surgery for vestibular schwannoma.^1–3^ In case of laceration during the transmeatal stage of resection, all actions should be focused on preventing air embolism. The defect may be effectively closed with a crushed muscle patch, and extreme care must be taken to position the graft without occluding blood flow. It is also prudent to evaluate stopping surgery in case complete occlusion is suspected, particularly in dominant bulbs, as severe cerebellar edema may rapidly set in and further complicate resection. In nondominant bulbs, occlusion may produce engorgement of cerebellar veins, or an increase in cerebellar venous bleeding. Finally, anticoagulation is not usually indicated, and few reports have shown safety in close monitoring of these cases.^4–6^

## Disclosures

The authors report no conflict of interest concerning the materials or methods used in this study or the findings specified in this publication.

## Author Contributions

Primary surgeon: Ramina. Assistant surgeon: Constanzo, Coelho Neto. Editing and drafting the video and abstract: Constanzo, Teixeira. Critically revising the work: Teixeira, Coelho Neto, Ramina. Reviewed submitted version of the work: all authors. Approved the final version of the work on behalf of all authors: Constanzo. Selecting and editing neuroimages: Teixeira.
